# Supplementary material for: Single Cell Transcriptomic and Chromatin Profiles Suggest Layer Vb Is the Only Layer With Shared Excitatory Cell Types in the Medial and Lateral Entorhinal Cortex
Source: Front Neural Circuits. 2022 Jan 26;15:806154. doi: 10.3389/fncir.2021.806154 (PMC8826650; doi:10.3389/fncir.2021.806154)
Supplement: Supplementary file 14 [file Data_Sheet_1.ZIP › Exc_vs_Inh_Motifs/homerResults/motif12.similar.html]

motif12

## Information for motif12

C
T
G
A
C
G
A
T
A
G
T
C
G
C
A
T
T
C
G
A
G
A
C
T
G
A
T
C
G
A
C
T
G
C
T
A
G
A
C
T
A
G
T
C
G
A
C
T
  
Reverse Opposite:  

C
G
T
A
T
C
A
G
C
T
G
A
C
G
A
T
C
T
G
A
C
T
A
G
C
T
G
A
A
G
C
T
C
G
T
A
T
C
A
G
G
C
T
A
G
A
C
T
  

|  |  |
| --- | --- |
| p-value: | 1e-186 |
| log p-value: | -4.290e+02 |
| Information Content per bp: | 1.817 |
| Number of Target Sequences with motif | 25152.0 |
| Percentage of Target Sequences with motif | 59.62% |
| Number of Background Sequences with motif | 5714.0 |
| Percentage of Background Sequences with motif | 52.57% |
| Average Position of motif in Targets | 377.1 +/- 260.0bp |
| Average Position of motif in Background | 339.0 +/- 261.6bp |
| Strand Bias (log2 ratio + to - strand density) | 0.2 |
| Multiplicity (# of sites on avg that occur together) | 1.67 |
| Motif File: | file (matrix) reverse opposite |

### Similar de novo motifs found

|  |  |  |  |  |  |  |  |
| --- | --- | --- | --- | --- | --- | --- | --- |
| Rank | Match Score | Redundant Motif | P-value | log P-value | % of Targets | % of Background | Motif file |
| 1 | 0.635 | C A T G T G C A A C T G C G T A G A C T T C G A C A T G C T G A | 1e-164 | -378.546333 | 53.01% | 46.36% | motif file (matrix) |
